# Relative displacement of anastomotic site of pancreato-jejunostomy in pancreatico-duodenectomy: a novel surgical reconstructive technique

**DOI:** 10.1186/1752-1947-7-209

**Published:** 2013-08-14

**Authors:** Teruyuki Usuba, Toshio Iino, Nobuyoshi Hanyu

**Affiliations:** 1 Department of Surgery, Machida Municipal Hospital, Asahicho 2-15-41, Machida-shi, Tokyo 194-0023, Japan

**Keywords:** Displacement of anastomosis, pancreatic fistula, pancreatico-duodenectomy

## Abstract

**Introduction:**

Intra-abdominal hemorrhage following pancreatic fistula is a fatal complication after pancreatico-duodenectomy. Intra-abdominal hemorrhage has reportedly decreased with the use of fibrin glue or polyglycolic acid felt and wrapping of the skeletonized vessels by omentum or falciform ligament. However, there are no extremely effective methods for the prevention of hemorrhage. Here, we report our novel and simple method for the prevention of intra-abdominal hemorrhage due to pancreatic fistula.

**Methods:**

The anastomotic site of the pancreato-jejunostomy in pancreatico-duodenectomy is displaced from the superior to inferior side of the transverse mesocolon through a small window created on the left side of the middle colic artery of the transverse mesocolon. This procedure is expected to prevent exposure of the skeletonized vessels to activated pancreatic juice from a pancreatic fistula after lymph node dissection, decreasing the incidence of hemorrhage. Two drains are placed on the superior and inferior sides of the transverse mesocolon. We performed this procedure in seven patients and compared the amylase level in the drainage fluid from the superior and inferior sides.

**Results:**

There was no difference in the fluid amylase level from the drains between the superior and inferior sides, because a pancreatic fistula was not present in all our patients. Therefore, we could not evaluate the efficacy of this method in the current study.

**Conclusions:**

Our procedure is theoretically expected to prevent intra-abdominal hemorrhage and will be an option in pancreatico-duodenectomy, especially for patients with a soft pancreas. However, it is necessary to evaluate the performance and results of this procedure in many more patients.

## Introduction

The post-operative morbidity and mortality rates of pancreatico-duodenectomy are higher than those of other procedures. In particular, pancreatic fistula following anastomotic leakage after pancreato-jejunostomy is a severe complication with high mortality. The skeletonized vessels following lymph node dissection are exposed to activated pancreatic juice from anastomotic leakage after pancreato-jejunostomy, resulting in pseudoaneurysm formation and intra-abdominal hemorrhage. The mortality rate is reported to range from 1 percent to 5 percent [1-6]. However, because of advances in interventional techniques, mortality due to intra-abdominal hemorrhage has decreased. Moreover, the use of fibrin glue or polyglycolic acid felt and wrapping of the skeletonized vessels by omentum or falciform ligament reportedly decreases intra-abdominal hemorrhage [7-15]. However, intra-abdominal hemorrhage remains a frequent complication, and many surgeons are concerned by this complication. Here we present a novel and simple technique for prevention of intra-abdominal hemorrhage due to pancreatic fistula after pancreatico-duodenectomy.

## Methods

**Figure 1:**
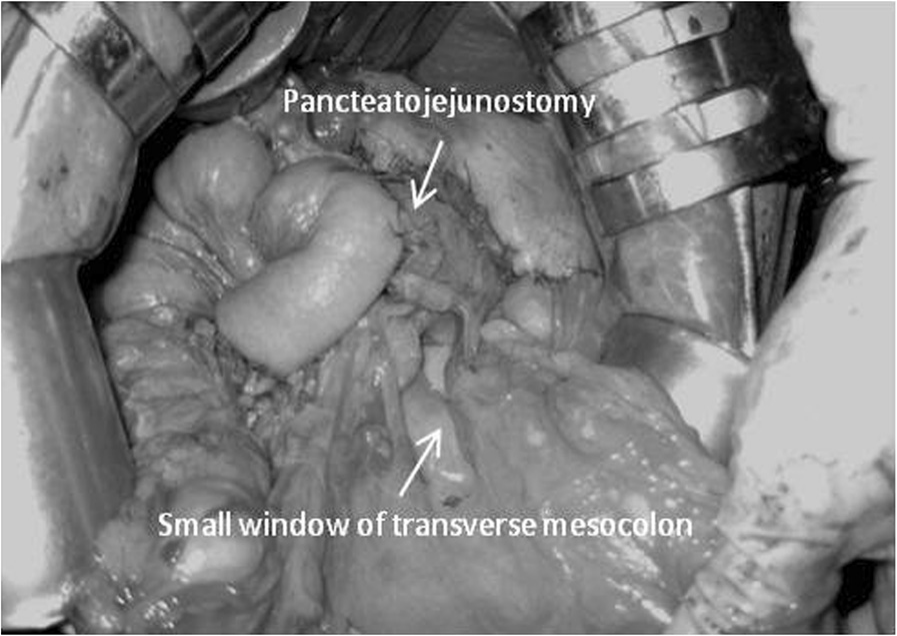
A small window is created on the left side of the middle colic artery of the transverse mesocolon.

**Figure 2:**
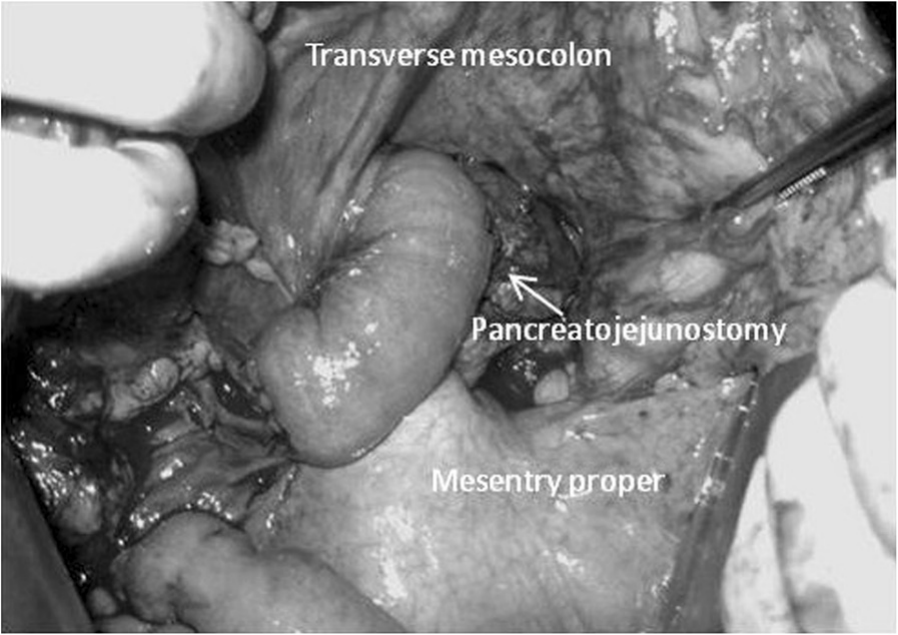
The anastomotic site of pancreato-jejunostomy is relatively displaced to the inferior side of the transverse mesocolon.

**Figure 3:**
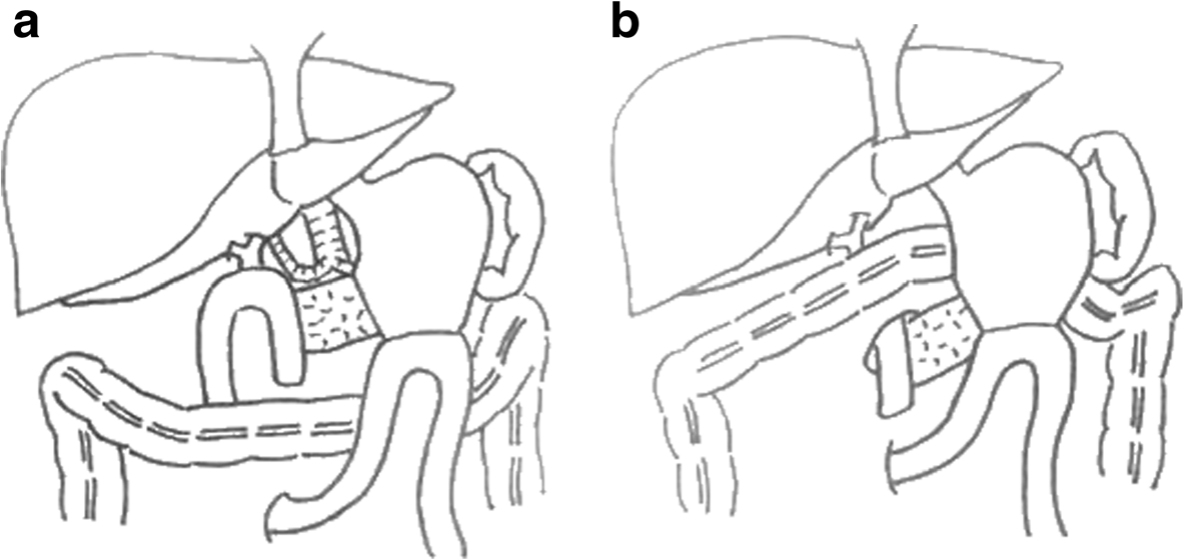
**The schema of reconstructions. (a)** Conventional reconstruction. **(b)** Our method.

Our procedure first involves reconstruction with a duct-to-mucosa pancreato-jejunostomy, continual end-to-side hepato-jejunostomy, and end-to-side gastro-jejunostomy. After all reconstructions have been performed, a small window is created on the left side of the middle colic artery of the transverse mesocolon (Figure 1). The anastomotic site of the pancreato-jejunostomy is then displaced from the superior to the inferior side of the mesocolon through this window. Finally, this window is closed with a few sutures. The anastomotic site is relatively displaced to the inferior side of the transverse mesocolon (Figure 2). Two closed drains are placed, one on the superior side of the mesocolon and the other on the inferior side near the anastomotic site. The schema of this procedure is illustrated in Figure 3.

We used this method in seven patients with a mean age of 70 years; six of our patients were men, with one woman. The primary diseases were intra-ductal papillary mucinous neoplasm (three patients), ampullary carcinoma (two patients) and tumor-forming pancreatitis (two patients). Fibrin glue was not used in any of our patients, and they were followed up for more than one year after the operation.

We examined the fluid amylase from the two drains three days after the operation for all our patients and compared the levels between the superior and inferior sides. Pancreatic fistula was classified in accordance with the International Study Group Pancreatic Fistula (ISGPF) definition [6]. Comparisons between the two groups were conducted using the Student’s t-test. *P* values of <0.05 were judged to be statistically significant.

## Results

The mortality and morbidity rates were 0 percent and 14 percent, respectively. One patient developed a grade A pancreatic fistula, and none of the remaining patients developed grade B or C pancreatic fistulas. Intra-abdominal hemorrhage did not appear in any of our patients. The average fluid amylase level of the superior and inferior sides was 109IU/L (range 4 to 310IU/L) and 343IU/L (range 19 to 1433IU/L), respectively. There was no significant difference in the fluid amylase levels between the superior and inferior sides. The average post-operative hospital stay of our seven patients was 16.5 days (range 10 to 38 days).

## Discussion

Pancreatico-duodenectomy is one of the most difficult procedures for gastroenterological surgeons, and has high morbidity and mortality rates. Pancreatic fistula is a common and severe complication after pancreatico-duodenectomy and often causes pseudoaneurysm formation and intra-abdominal hemorrhage. Hemorrhage can occur not only in the peri-operative period, but also a long time after the operation.

It is reported that the mortality rate of intra-abdominal hemorrhage formerly ranged from 30 percent to 58 percent, however it has recently decreased to less than 5 percent because the trans-arterial embolization technique has progressed [1-6,16-19]. Furthermore, it was reported that the combination of polyglycolic acid felt and fibrin glue prevents pancreatic fistula after pancreatico-duodenectomy and that wrapping the major vessels using the falciform ligament or omentum decreases the incidence of pancreatic fistula and intra-abdominal hemorrhage [11-19]. However, the mortality rate after pancreatico-duodenectomy is still higher than that after other procedures. For patients with pancreatic head carcinoma, a part of the transverse mesocolon is resected for lymph node and nerve plexus dissection, while the transverse mesocolon is left in place in patients with ampullary carcinoma, intra-ductal papillary mucinous neoplasm, or tumor-forming pancreatitis because these diseases do not require extensive dissection of the lymph nodes or para-superior mesenteric artery. Because the physical quality of most pancreata with pancreatic carcinoma is hard, the risk of pancreatic fistula in patients with pancreatic carcinoma is lower than that in patients with a soft pancreas. Therefore, pancreatic carcinoma is excluded from the indications for our procedure. It has been reported in the Japanese literature that displacing the stump of the remnant pancreas from the superior to inferior side of the transverse mesocolon after total gastrectomy with distal pancreatectomy and splenectomy prevented intra-abdominal hemorrhage due to pancreatic fistula. This report demonstrated that the fluid amylase level of the superior side was less than that of the inferior side, and as a result, intra-abdominal hemorrhage deceased. By applying this technique and theory to pancreatico-duodenectomy, we developed a method involving relative displacement of the anastomotic site of the pancreato-jejunostomy. The anastomotic site of the pancreato-jejunostomy is originally located on the superior side of the transverse mesocolon, therefore the skeletonized vessels, such as the common hepatic artery, proper hepatic artery, and stump of the gastro-duodenal artery, are exposed to pancreatic juice when a pancreatic fistula is present. By displacing the anastomotic site from the superior to inferior side, these arteries are not exposed to the pancreatic juice, preventing intra-abdominal hemorrhage. We did not examine whether pancreatic juice seeped through the mesentery, but our procedure was developed on the condition that pancreatic juice does not seep.

Because most of our patients did not develop a pancreatic fistula, their post-operative outcomes were not improved by performing our procedure.

This relative displacement of the anastomotic site of the pancreato-jejunostomy in pancreatico-duodenectomy may prevent intra-abdominal hemorrhage due to pancreatic fistula formation after pancreatico-duodenectomy. Notably, this procedure should be performed in patients with a soft pancreas. However, we only used this procedure with a few of our patients; moreover, there were no incidents of severe pancreatic fistula or intra-abdominal hemorrhage. Many more patients with severe pancreatic fistula are needed to confirm the efficacy of our procedure.

## Conclusions

Relative displacement of the anastomotic site of the pancreato-jejunostomy may be expected to prevent intra-abdominal hemorrhage and will be an option in pancreatico-duodenectomy.

## Consent

Written informed consent was obtained from the patients for publication of this manuscript and any accompanying images. A copy of written consent is available for review by the Editor-in-Chief of this journal. Ethical approval for this research study was obtained from our local Institutional Review Board.

## Competing interests

The authors declare that they have no competing interests.

## Authors’ contributions

TU and TI drafted the manuscript. TU contributed to the data collection and NH supervised manuscript writing. All authors read and approved the final manuscript.
